# A Short Synthesis of Bisabolane Sesquiterpenes

**DOI:** 10.3390/molecules16098053

**Published:** 2011-09-19

**Authors:** Zhen-Ting Du, Shuai Zheng, Gang Chen, Dong Lv

**Affiliations:** 1 College of Science, Northwest A&F University, Yangling, Shaanxi 712100, China; Email: 379746048@qq.com (S.Z.); 405984656@qq.com (G.C.); 251200758@qq.com (D.L.); 2 Key Laboratory of Protection and Utilization of Biological Resources in Tarim Basin, Talimu University, Alaer, Xinjiang 843300, China

**Keywords:** curcumene, xanthorrhizol, curcuhydroquinone, synthesis

## Abstract

A facile total synthesis of three members of the bisabolane sesquiterpene family, namely (±)-curcumene, (±)-xanthorrhizol and (±)-curcuhydroquinone had been achieved in high overall yield. The synthesis used bromobenzene derivatives as starting materials. The halogen-lithium exchange followed by addition of isoprenylacetone and reduction of the obtained carbinols are the key steps of the synthetic pathway. This synthetic approach provides a new route to the bisabolane sesquiterpenes.

## 1. Introduction

**Figure 1:**
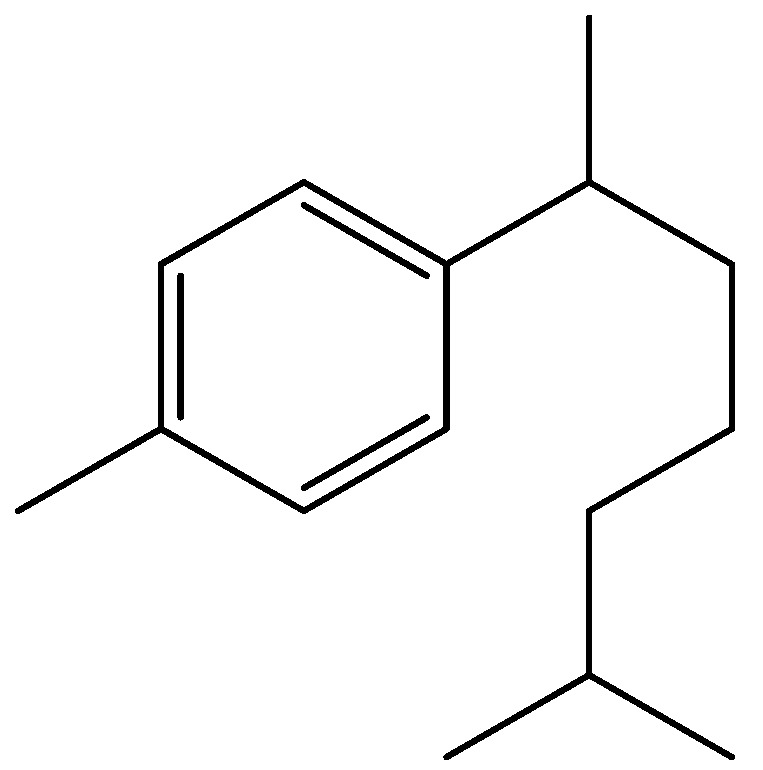
Aromatic bisabolane sesquiterpene skeleton.

**Scheme 1:**
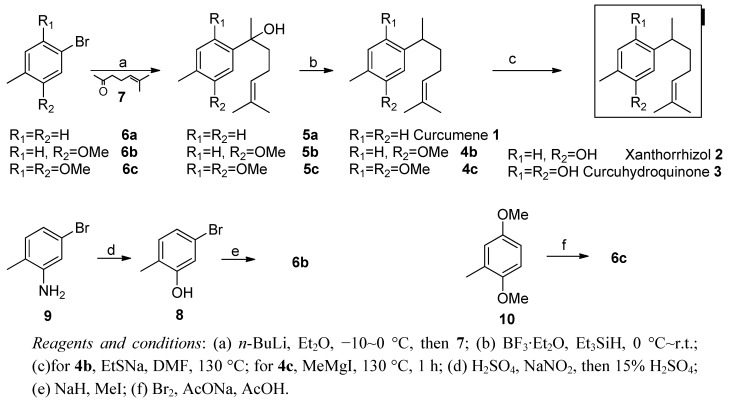
Synthesis of Curcumene **1**, Xanthorrhizol **2** and Curcuhydroquinone **3**.

Among the various important classes of natural products, the bisabolane sesquiterpenes [1,2,3,4,5] refer to the C_15_ compounds which include a mono-aromatic ring skeleton (Figure 1). Among them, curcumene is the simplest aromatic one, and (+)-α-curcumene (**1**, Scheme 1) has been recognized as a constituent of the essential oil from the rhizomes of *Curcuma aromatica *by Simonsen and a number of essential oils [1].

Xanthorrhizol (**2**, Scheme 1), bearing only one phenolic hydroxyl group *ortho* to the methyl group, was firstly discovered from *Curcuma xanthorrhiza*, which was used as traditional medicine in Indonesia [6,7]. Afterward, xanthorrhizol (**2**) was isolated from the same plant again as an antitumor compound by Itokawa [8].

Xanthorrhizol (**2**) exhibits antibacterial activity against *Streptococcus mutans*, anticarcinogenic activity, as well as suppressing activity of cyclooxygenase-2 and inducible nitric oxide synthase enzyme which are related to the inflammation process. Moreover, xanthorrhizol was found to prolong pentobarbital-induced sleeping time, an effect due to inhibition of cytochrome P-450 activity [9,10,11,12]. Sirat reported synthesis of several bisabolane sesquiterpenoids using xanthorrhizol as a building block [13]. (−)-Curcuhydroquinone (4, Scheme 1) was isolated from the Caribbean gorgonian *Pseudopterogorgia rigida* and showed antibacterial activity against *Staphylococcus aureus* and the marine pathogen *Vibro anguillarum* [14]. Curcuhydroquinone can be used to synthesize several heliannuols [15,16], which are a class of natural allelochemicals. Because of its wide range of biological activities and its synthetical utility, this kind of naturally occurring products are attractive synthetic targets, especially to verify the usefulness of newly developed synthetic methodology. Some syntheses of curcumene (**1**), xanthorrhizol (**2**) and curcuhydroquinone (**3**) have been reported [17,18,19,20,21,22,23,24,25,26,27,28,29,30,31,32,33,34,35,36,37,38,39]; however, they may suffer from some drawbacks, such as the use of toxic materials, commercially unavailable reagents or starting materials, too many chemical operations or limited applicability. In our synthetic approaching of heliannuols mentioned above, there was a need for a quick and labor-saving route to these compounds or their analogues as building blocks. Therefore, there is still a perceived demand for development of a practical synthetic route to them.

One of the most-used methods to construct C–C bonds is through addition of lithium reagents to carbonyls. As an extension of our continuous effort to synthesize those kinds of naturally occurring products and to test their potential application in agrochemicals [40,41,42,43,44], we wish to report herein a new and efficient total synthesis of curcumene (**1**), xanthorrhizol (**2**), and curcuhydroquinone (**3**) using cheap starting materials, where lithium reagent addition and selective carbinol reduction were used as key steps. The salient feature of our synthetic approach is that it combines two well-known chemical procedures.

## 2. Results and Discussion

As shown in Scheme 1, our synthesis is designed as a C7 + C8 strategy. Based the works of Boukouvalas [45], the connection step was mediated through a lithium reagent. It commenced from bromobenzene derivatives **6a–6c**. Because of the derivatives of bromobenzene **6b** are not commercially available, 4-bromo-2-aminotoulene (**9**) was diazotized, hydrolyzed [46] and then methylated to give **6b**. Compound **6c** was prepared in high yields according to the literature [47,48]. In order to construct the skeleton of our molecules, the alcohols **5a–5c** can be obtained by the addition reaction between lithium reagent of compound **6a–6c** and isoprenylacetone **7**. With three key intermediates in hand, the alcohols **5a–5c** were selectively reduced at the presence of BF_3_·Et_2_O/Et_3_SiH [23] to give curcumene (**1**), **4b** and **4c** respectively. (±)-Xanthorrhizol (**2**) and (±)-curcuhydroquinone (**3**) were obtained after a conventional demethylation protocol of the corresponding methyl ethers **4b** and **4c**, respectively. It is obvious that only three chemical operations are needed in the synthesis of curcumene (**1**). As far as the synthesis of (±)-xanthorrhizol (**2**) is concerned, our approach is still effective. In this way, we could accumulate large quantities of natural products, namely curcumene (**1**), xanthorrhizol (**2**) and curcuhydroquinone (**3**) in a short time.

## 3. Experimental Section

### General

^1^H and ^13^C-NMR data were recorded in CDCl_3_ with Bruker-500 spectrometer if not noted otherwise. The chemical shifts were reported in ppm relative to TMS. Mass spectra were recorded on a HP-5988 mass spectrometer (EI), Thermo Scientific LCQ-FLEET (ESI) or Bruker FT-MS analyzer (HRMS), respectively. Column chromatography were generally performed on silica gel (200–300 mesh) eluting with petroleum ether-EtOAc (100:1–10:1 v/v) and TLC inspections on silica gel GF254 plates with petroleum ether-EtOAc (20:1–5:1 v/v) if not noted otherwise.

*5-Bromo-2-methylphenol methyl ether *(**6b**). A mixture of 5-bromo-2-methylaniline (8 g, 42.9 mmol) and sulfuric acid (48 mL) in water (600 mL) was heated to 120 °C for 3 h and then the reaction mixture was cooled with ice bath. NaNO_2_ (3.0 g, 44.6 mmol) was added portionwise at 0 °C to the above suspension, which was monitored by KI/starch indicator. The reaction mixture was added potionwise to sulfuric acid (48 mL) in water (600 mL) which had been preheated to 90 °C, and the mixture was maintained for 1 h and then poured into ice to give crude 5-bromo-2-methylphenol (**8**, 4.2 g, 52%). Then a mixture of crude **8** (1.87g, 10 mmol), anhydrous THF (20 mL), NaH in mineral oil (600 mg, 15 mmol) and MeI (2.8 g 20 mmol), was refluxed for 4 h. The mixture was quenched, poured into water, extracted with ether, and the combined extracts was washed with saturated Na_2_CO_3_, water, brine successively, dried, and purified through column chromatography to give 5-bromo-2-methyl phenol methyl ether (**6b**, 1.7 g, 85%) as a colorless oil. ^1^H-NMR: 2.36 (s, 3H), 3.84 (s, 3H), 6.39 (s, 1H), 6.78 (d, *J *= 7.6 Hz, 1H), 7.20 (d, *J *= 7.6 Hz, 1H).

*6-Methyl-2-(p-tolyl)hept-5-en-2-ol *(**5a**). At −20 °C, to a solution of 4-bromotoulene (500 mg, 2.9 mmol) in anhydrous THF (10 mL) was added BuLi (1.46 mL, 2.2 M) dropwise, and the reaction mixture was stirred for half an hour. The mixture was cooled to −78 °C, then isoprenylacetone (360 mg, 2.9 mmol) in anhydrous THF (10 mL) was added dropwise. Then the reaction mixture was stirred for 2 h at −40 °C, and then quenched with saturated NH_4_Cl, and extracted with ether (30 mL × 3). The combined organic layers were washed with brine, dried, filtered and evaporated under reduced pressure to afford crude product. Purification on silica gel yielded 6-methyl-2-(*p*-tolyl)-hept-5-en-2-ol (531 mg, 84%) as a viscous oil. ^1^H-NMR: 1.49 (s, 3H), 1.52 (s, 3H), 1.65 (s, 3H), 1.81–1.98 (m, 5H), 2.33 (s, 3H), 5.08 (t, *J *= 6.5 Hz, 1H), 7.14 (d, *J *= 8.0 Hz, 2H), 7.30 (d, *J *= 8.0 Hz, 2H). ^13^C-NMR: 17.6, 20.9, 23.0, 25.79, 30.5, 43.7, 74.8, 124.3, 124.7, 128.8, 132.1, 136.0, 145.0. ESP-MS: M+H−H_2_O = 201.

*2-(3-Methoxy-4-methylphenyl)-6-methylhept-5-en-2-ol* (**5b**). The method was identical to that described for the preparation of **5a**. ^1^H-NMR: 1.43 (s, 3H), 1.46 (s, 3H), 1.65 (s, 3H), 1.91–2.04 (m, 4H), 2.17 (s, 3H), 3.84 (s, 3H), 4.92 (br s, 1H), 5.11 (t, *J *= 6.5 Hz, 1H), 6.30 (d, *J *= 8.0 Hz, 1H), 6.38 (s, 1H), 6.94 (d, *J *= 8.0 Hz, 1H). ^13^C-NMR: 10.7, 12.9, 18.7, 19.8, 21.0, 25.0, 34.4, 50.5, 63.4, 93.8, 101.5, 113.8, 119.3, 125.9, 127.4, 150.1, 153.9. ESP-MS: M+H−H_2_O = 231.

*2-(2,5-Dimethoxy-4-methylphenyl)-6-methylhept-5-en-2-ol *(**5c**). The method was identical to that described for the preparation of **5a**. ^1^H-NMR: 1.52 (s, 3H), 1.56 (s, 3H), 1.65 (s, 3H), 1.81–1.98 (m, 4H), 2.21 (s, 3H), 3.80 (s, 3H), 3.82 (s, 3H), 3.94 (br, s, 1H), 5.09 (t, *J *= 6.5 Hz, 1H), 6.71 (s, 1H), 6.83 (s, 1H). ^13^C-NMR: 15.9, 17.5, 23.3, 25.6, 27.6, 42.0, 55.9, 56.0, 75.1, 109.8, 114.5, 124.6, 125.6, 131.4, 132.8, 150.3, 151.5. ESP-MS: M+H−H_2_O = 261.

*Curcumene* (**1**). At 0 °C, to a solution of **5a** (0.4 g, 1.83 mmol) in dry dichloromethane (15 mL), triethylsilane (0.25 g, 2.2 mmol), was added boron trifluoride diethyl etherate (0.27, 1.9 mmol) with stirring. The mixture was stirred for half an hour, quenched with a few drops of water, and extracted with dichloromethane (20 mL × 3). The combined organic solutions were dried and evaporated. The residue was purified through silica gel column chromatography to give curcumene (**1**, 347 mg, 94%) as a colorless oil. IR(film/cm^−1^): 2,962, 2,923, 2,857, 1,516, 1,453, 1,376, 8,16; ^1^H-NMR: 1.21 (d, *J* = 7 Hz, 3H), 1.53 (s, 3H), 1.60–1.67 (m, 2H), 1.68 (s, 3H), 1.80–1.95 (m, 2H), 2.32 (s, 3H), 2.55–2.65 (m, 1H), 5.10 (t, *J* = 6.9 Hz, 1H), 7.03 (d, *J* = 7.8 Hz, 2H), 7.08 (d, *J* = 7.8 Hz, 2H). ^13^C-NMR: 17.6, 20.9, 22.4, 25.7, 26.1, 38.5, 39.0, 124.6, 126.9, 129.0, 131.3, 135.1, 144.6. MS (EI): 202 (M+, 27), 187 (4), 159 (22), 145 (27), 132 (76), 119 (100).

*2-Methyl-6-(3-methoxy-4-methylphenyl)-heptene* (**4b**). The method was identical to that described for the preparation of **1** (colorless oil, 93%). IR (film/cm^−1^): 2,958, 2,922, 1,612, 1,581, 1,508, 1,459, 1,414, 1,256, 1,135, 1,043, 851, 815. ^1^H-NMR (500 MHz, CDCl_3_): 1.23 (3H, d, *J *= 7.0 Hz), 1.51 (3H, s), 1.51–1.65 (2H, m), 1.66 (3H, s), 1.86–1.92 (2H, m), 2.18 (3H, s), 2.63–2.67 (1H, m), 3.82 (3H, s), 5.10 (1H, t, *J *= 7.1 Hz), 6.65 (1H, d, *J *= 1.1 Hz), 6.68 (1H, dd, *J *= 7.55 Hz, *J *= 1.45 Hz), 7.04 (1H, d, *J *= 7.55 Hz). ^13^C-NMR (125 MHz, CDCl_3_): 16.0 (CH_3_), 17.9 (CH_3_), 22.7 (CH_3_), 25.9 (CH_3_), 26.4 (CH_2_), 38.6 (CH_2_), 39.7 (CH), 55.4 (CH_3_), 109.1 (CH), 118.8 (CH), 124.7 (CH), 124.8 (CH), 130.5 (CH), 131.5(C), 146.9 (C), 157.8 (C). ESP-MS: M+H = 233.

*2-Methyl-6-(2,5-dimethoxy-4-methylphenyl)-heptene* (**4c**). The method was identical to that described for the preparation of **1** (94% yield as a colorless oil). ^1^H-NMR: 1.28 (d, *J *= 7.2Hz, 3H), 1.56 (s, 3H), 1.57–1.59 (m, 2H), 1.75 (s, 3H), 1.96–2.04 (m, 2H), 2.33 (s, 3H), 3.21–3.26 (m, 1H), 3.87 (s, 3H), 3.88 (s, 3H), 5.22 (t, *J *= 6.9Hz, 1H), 6.67 (s, 1H), 6.70 (s, 1H). ^13^C-NMR: 16.0, 17.5, 21.2, 25.6, 26.3, 31.8, 37.3, 56.0, 56.3, 111.6, 112.5, 122.0, 125.0, 126.7, 133.1, 136.8, 157.1. EIMS (*m/z*): 262, 247, 219, 192, 179, 149, 119, 91. HRMS: required C_17_H_27_O_2_ 262.2013, found 262.2009.

*Xanthorrhizol* (**2**). To a solution of EtSH (0.8 mL, 10.82 mmol) in anhydrous DMF (35 mL) was added NaH (60% in mineral oil, 430 mg, 10.82 mmol), then compound **4b** (500 mg, 2.15 mmol) in DMF (2 mL) was added, and the mixture was refluxed for 6 hours. After completion, the mixture was poured into ice water, and was acidified, and extracted. The extraction was condensed, and the residue was purified by common column chromatography to give xanthorrhizol (**2**, 410 mg, 87%). IR (cm^−1^): 3,392, 2,960, 2,922, 1,585, 1,453, 1,256, 1,176, 1,122, 880, 815. ^1^H-NMR (500 MHz, CDCl_3_): 1.18 (3H, d, *J *=7.0 Hz), 1.52 (3H, s), 1.51–1.65 (2H, m), 1.66 (3H, s), 1.86–1.92 (2H, m), 2.20 (3H, s), 2.56–2.63 (1H, m), 4.66 (1H, br, s), 5.06–5.09 (1H, m), 6.59 (1H, d, *J *= 1.5 Hz), 6.60 (1H, dd, *J *= 7.7 Hz, *J *= 1.50 Hz), 7.02 (1H, d, *J *= 7.7 Hz). ^13^C-NMR (125 MHz, CDCl_3_): 15.5 (CH_3_), 17.8 (CH_3_), 22.5 (CH_3_), 25.8 (CH_3_), 26.3 (CH_2_), 38.5 (CH_2_), 39.2 (CH), 113.7 (CH), 119.6 (CH), 120.9 (C), 124.7 (CH), 130.9 (CH), 131.6 (C), 147.4 (C), 153.7 (C). ESP(−)-MS: M−H = 217. HRMS: required C_15_H_22_O_2_, 218.1671, found 218.1673.

(±)-*Curcuhydroquinone* (**3**). To a suspension Mg (1.45 g, 60 mmol) in 10 mL of anhydrous diethyl ether was added slowly iodomethane (6.35 g, 45 mmol) in anhydrous diethyl ether at room temperature. After completion, the reaction mixture was refluxed for 1 h and then the mixture was evaporated in vacuum. The neat compound **4c** (393mg, 1.5 mmol) was heated to 180 °C for 15 min, after cooled to room temperature and 100 mL 1.0 M HCl was added to quench the reaction. The mixture was extracted EtOAc (3 × 20mL); the combined organic layer was washed with brine and dried over Na_2_SO_4_. After evaporation the residue was purified through column chromatography to give **3** (316 mg, 90%) as a colorless oil. IR (neat): 3,545, 2,966, 2,927, 1,666, 1,615, 1,456, 1,417, 1,377, 1,306, 1,187, 1,003, 875, 833 cm^−1^. ^1^H-NMR: 1.20 (d, *J* = 7.2Hz, 3H), 1.53 (s, 3H), 1.50–1.63 (m, 2H), 1.68 (s, 3H), 1.92–1.95 (m, 2H), 2.17 (s, 3H), 2.94 (m, 1H), 4.65 (br, 1H), 5.15 (br, 1H), 6.56 (br, 1H), 6.71(br, 1H), 7.02 (d, *J* = 7.9Hz, 1H). ^13^C-NMR (CDCl_3_, 300 MHz): 15.4, 17.7, 21.1, 25.7, 26.0, 31.5, 37.4, 113.5, 118.0, 121.8, 124.6, 131.8, 132.1, 146.7, 147.8. MS (EI): 235 (M^+^+1), 234, 217, 191, 177, 164, 151, 137, 124, 107, 95, 77. HRMS: required C_15_H_22_O_2_, 234.1700, found 234.1703.

## 4. Conclusions

In summary, we have reported an effective and facile route for the synthesis of (±)-curcumene (**1**), (±)-xanthorrhizol (**2**) and (±)-curcuhydroquinone (**3**) using cheap starting materials. Our approach can provide large quantities of these versatile natural products using simple starting materials. The combination of lithium reagent addition and selective reduction by BF_3_^.^Et_2_O/Et_3_SiH were the key features of our approach.
